# Properties of Poly-γ-Glutamic Acid Producing-*Bacillus* Species Isolated From *Ogi* Liquor and Lemon-*Ogi* Liquor

**DOI:** 10.3389/fmicb.2019.00771

**Published:** 2019-04-16

**Authors:** Titilayo A. Ajayeoba, Stanley Dula, Oluwatosin A. Ijabadeniyi

**Affiliations:** Department of Biotechnology and Food Technology, Durban University of Technology, Durban, South Africa

**Keywords:** poly-γ-glutamic acid, *Bacillus*, *ogi* steep liquor, antimicrobial, angiotensin-converting enzyme

## Abstract

Poly-γ-glutamic acid (γPGA) is a natural and promising biopolymer synthesized by *Bacillus* spp. during fermentation. This study isolated *Bacillus* spp. from *ogi* steep liquor (OSL) and lemon-*ogi* steep liquor (LOSL) using standard methods and determined the γPGA-producing ability. The antimicrobial and angiotensin-converting enzyme (ACE) inhibitory activities of γPGA polymer were evaluated and isolates were sequenced. Four isolates (TA004, TA006, TA011, TA012) selected based on phenotypic characterization and stickiness (<15 cm) showed antibacterial activity against different pathogens with the highest activity found in TA004 (22.5 mm) and least in TA011 (16.6 mm). Furthermore, time-kill assay showed that the combined γPGA polymer was more effective and demonstrated bactericidal activity over individual γPGA which are bacteriostatic in nature. All γPGA polymer exhibited ACE properties except TA011. The highest IC_50_ was observed in TA006 (0.11 mg/ml) and least in TA004 (0.35 mg/ml). TA004 had the highest molecular weight (261 kDa) while TA011 had the least (194.97 kDa). In addition, all γPGA exhibited characteristic peaks at 3413–3268 cm^-1^ and 1722–1664 cm^-1^ that corresponded to amine N–H stretching intensities and C = O stretching in COOH. *Bacillus* isolates were identified as TA004 (*B. subtilis*-GenBank: MH782061), TA006 (*B. amyloliquefaciens*- GenBank: MH782075), TA011 (*B. subtilis*- GenBank: MH782088), TA012 (*B. subtilis*- GenBank: MH782083). OSL and LOSL have the potential for developing functional foods with a valuable effect on health.

## Introduction

Food and beverages can be fermented naturally and the product usually contains both beneficial and non-beneficial microorganisms. The beneficial microorganisms transform the composition of food materials, thereby enhancing the bio-availability of nutrients, degrading toxins and anti-nutritional constituents, stimulates probiotic functions, increase health-promoting bioactive compounds and may produce biopolymers under certain conditions (Baschali et al., 2017). Fermented whole cereal products have associated functional properties of reducing cholesterol, anti-inflammatory properties, improving antioxidant and vascular function (Jonnalagadda et al., 2011). Depending on the food substrate and condition of fermentation, strains of non-pathogenic *Bacillus* and *Lactobacillus* spp. are known for producing antimicrobial properties and biopolymers such as polyhydroxyalkanoates, polylactic, and polyglutamic acid.

Polyglutamic acid (PGA), a poly amino acid produced by *Bacillus* spp. is a naturally occurring anionic, water-soluble, biodegradable, non-toxic, viscous, edible biopolymer containing D and L-glutamic acid residues with different industrial applications (Bajaj and Singhal, 2011; Ogunleye et al., 2015). Poly γ-glutamic acid (γPGA), a type of PGA is majorly produced by *Bacillus subtilis, B. amyloliquefaciens, B. megaterium*, and *B. licheniformis* (Bajaj and Singhal, 2011; Ogunleye et al., 2015; Chettri et al., 2016). γPGA stimulates and improves immune activity, thus, has functionality for selective delivery of chemotherapeutic agents (Tork et al., 2015).

*Bacillus* spp. have been associated with some naturally fermented foods. These food products include *Kinema* (soybean), *Poto poto* and *dégué* (maize and millet), *Fufu, Gari, Lafun* (cassava), *Ogi* among others (Abriouel et al., 2006; Chettri et al., 2016; Tamang et al., 2016). *Ogi* is made from a variety of cereals like maize (*Zea mays*), sorghum (*Sorghum vulgare*), millet (*Pennisetum glaucum*) or its combination, but the steep liquor is usually wasted. *Ogi* steep liquor (OSL) is sometimes administered orally to influence gastrointestinal microflora and suppress the growth of pathogens (Evans et al., 2013) or combined with other antimicrobial substances such as lemon juice for the antimicrobial properties (Ajayeoba et al., 2016).

Not all chronic diseases are caused by pathogens; some are as a result of an individual’s lifestyle. Poor dieting, smoking, stress can also lead to a variety of chronic diseases such as hypertension, diabetes and cancer. The incidence of high blood pressure is becoming prevalent, thus posing a great challenge to human health (Shafieyan et al., 2016). Angiotensin-converting enzyme (ACE) plays a key role in the regulation of blood pressure by converting the inactive decapeptide angiotensin I and releasing into the octapeptide angiotensin II. This, in turn, exerts a strong vaso-constrictive action that inactivates vasodilators, increases artery pressure and ultimately leads to hypertension. This means ACE-inhibitors promotes antihypertensive action. Known potent ACE-inhibitors, such as captopril and lisinopril, have been used clinically to treat hypertension and heart failure in humans but there are reported side effects (Hernández-Ledesma et al., 2004).

Although the presence of *Bacillus* spp., lactic acid bacteria (LAB), and yeast have been reported at various stages of *Ogi* fermentation (Ijabadeniyi, 2007; Okeke et al., 2015), only some functional properties of the LAB are reported. A spectrum of *Bacillus* spp. has been identified in *Ogi* and OSL but potential benefits have not been reported. Furthermore, there are few reports on the microbial potentials of fermented cereal-based products. Since fermented products are known for potential antihypertensive and antimicrobial activities and reducing oxidative stress (Baschali et al., 2017), the microorganisms can be excellent resources for antimicrobial and ACE inhibitory effect (Iwaniak et al., 2014). While lemon juice-ogi steep liquor (LOSL) has been reported to have bioactive compounds, microorganisms having potential are yet to be explored. This study seeks to identify and evaluate some functional properties of γPGA producing *Bacillus* species from OSL and LOSL.

## Materials and Methods

### Sample Collection

White maize*-Zea mays*, White Sorghum-*Sorghum bicolor*, Pearl millet (*Pennisetum glaucum* L.) were purchased from Igbona market in Osogbo, Nigeria, while lemon (*Citrus limon*) fruits were purchased from Durban, South Africa. The γPGA sodium salt, media, chemical, and reagent were Oxoid products and Sigma-Aldrich grade.

### Traditional Fermentation of *Ogi* and Lemon Juice-*Ogi* Steep Liquor

The fermentation process of OSL and LOSL from different cereals followed the modified method described by Ajayeoba et al. (2016). Liquor samples of OSL and LOSL were taken from the unsieved *ogi* supernatant at every 6 h during a 72 h fermentation period to test for the presence of *Bacillus* species.

### Isolation of Microorganisms

Isolation of *Bacillus* spp. was carried out according to the modified method described by Chettri et al. (2016). About 10 ml of each liquor was homogenized with 90 ml sterile saline water in a stomacher (Fisher Scientific, United Kingdom) for 1 min. The mixture was serially diluted and heated at 100°C for 2 min, incubated for 24 h at 37°C and enumerated on sterile nutrient agar. Mucoid colonies were purified and preserved in 15% (v/v) glycerol at -80°C for further analysis.

### Phenotypic Characterization

The morphological and physiological characterization of the purified isolates were based on microscopic shape, Gram staining reaction, catalase, motility test, endospore formation, production of carbon dioxide from glucose, ammonia from arginine, growth at different temperatures (30 and 45°C), NaCl concentrations (1.5 and 2.5%) and pH in nutrient broth following the method of Schillinger and Lücke (1987). Furthermore, Voges– Proskauer test, nitrate reduction, starch hydrolysis, casein hydrolysis, citrate utilization test, bile salt tolerance, anaerobic growth, and sugar fermentations were determined following the method of Duc et al. (2004). Taxonomic key of Slepecky and Hemphill (2006) was followed for the identification of *Bacillus* spp.

### Screening of γPGA Producing *Bacillus* spp.

The stickiness of prospective *Bacillus* isolates was measured and screened for potentials of presumably γPGA production according to the modified described by Chettri et al. (2016). The precipitated γPGA was purified, dialyzed overnight against distilled water, freeze-dried and used to evaluate some functional properties of γPGA.

### Functional Properties of γPGA Polymer

#### Angiotensin-Converting Enzyme (ACE) Inhibitory Activity

The ACE inhibitory activities of each purified γPGA and inhibition concentration (IC_50_) values were evaluated according to the modified method described by Lee et al. (2014a). The hippuryl-histidyl-leucine (HHL) was dissolved in 100 mM phosphate buffer (pH 8.3) containing 300 mM NaCl. Then, 100 μl of HHL (12.5 mM) and 50 μl of the sample were pre-incubated at 37°C for 5 min. Hundred microliter of ACE dissolved in distilled water (0.01 U/ml) was added and incubated at 37°C for 30 min. The enzymatic reaction was terminated by adding 250 μl of 0.1 N HCl. The liberated hippuric acid was extracted from the acidified solution into 1.5 ml of ethyl acetate by vortex mixing for 15 s, and centrifuging for 10 min to separate the ethyl acetate layer. Five hundred microliter aliquot of supernatant was taken into another flask and evaporated on a heating block at 90°C for 1 h. The hippuric acid was redissolved in 1 ml distilled water and absorbance was measured at 228 nm. N-[(S)-mercapto-2-methylpropionyl]-L-proline (Captopril) was used as positive control. ACE inhibition activity was calculated using the following equation:

$$ACE\;inhibition\;activity\;(\%)=[\frac{1-AbsorbanceofSample}{AbsorbanceofControl}]\times 100$$

#### Antibacterial Activity

For the antimicrobial activity, the paper disc was impregnated with each γPGA (150 mg/ml) and tested against the following pathogenic microorganism: *Escherichia coli* ATCC 25922, *Staphylococcus aureus* ATCC 29213, *Salmonella enterica* ATCC 13314, *Pseudomonas aeruginosa* ATCC 27853, *Listeria monocytogenes* ATCC 7644, and *Bacillus cereus* ATCC 10876. Minimum inhibitory concentrations (MIC) were further carried out as described by Clinical and Laboratory Standards Institute [CLSI] (2012). MIC was the lowest concentration of the antimicrobial agent (γPGA solution) that completely inhibits the growth of the microorganism.

#### Time-Kill Kinetics Study

Time-kill assay of γPGA was carried out according to the recommended CLSI standard as described by Chen et al. (2016). Bacteriostatic effect of PGA was interpreted as < 3log_10_, while bactericidal effect as ≥ 3 log_10_ in viable colony relative to initial inoculum.

### Analysis of γPGA Polymer

The yield of each γPGA polymer was detected using spectrophotometric ultraviolet assay while polysaccharide and protein contents were determined using phenol-sulphuric acid and bicinchoninic acid as described by Zeng et al. (2012). Glucose and bovine serum albumin were used as standards. Furthermore, the molecular weights of γPGA were detected by measuring the diffusion distance of the concentric zone on plates according to Zeng et al. (2013). After incubating the plate at 37°C for 5 h, the distance observed was measured by a Vernier caliper and the molecular weight was calculated with the equation; (y = a + bx, where a = 10.607, b = -0.000677013, y = diffusion distance, x = molecular weight). Fourier Transform Infrared Spectroscopy (FT-IR) (PerkinElmer) was also used to determine organic groups in the range of 4000–400 cm^-1^ (Konglom et al., 2012).

### *16s rRNA* Sequencing of γPGA Producing Isolates

*16s rRNA* sequences of the selected isolates were identified by PCR at Inqaba Biotech, South Africa using 27F (5′-AGAGTTTGATCCTGGCTCAG-3′) and 1492R (5′-CGGTTACCTTGTTACGACTT-3′) primers (Altschul et al., 1997). PCR products were sequenced, assembled and edited with software BioEdit 7.2.5. Basic Local Alignment Search Tool (BLAST)^1^ was used to compare the consensus sequences with those deposited in the GenBank DNA database. A phylogenetic tree based on *16s rRNA* genes was constructed to determine the closest bacterial species by the neighbor-joining method (Saitou and Nei, 1987), using MEGA7 (Kumar et al., 2016). Sequence divergence among LAB isolates were quantified using the Maximum Composite Likelihood method (Tamura et al., 2004).

## Results and Discussion

### Isolation, Phenotypic Characterization of *Bacillus* spp. and Screening for PGA Production

**FIGURE 1:**
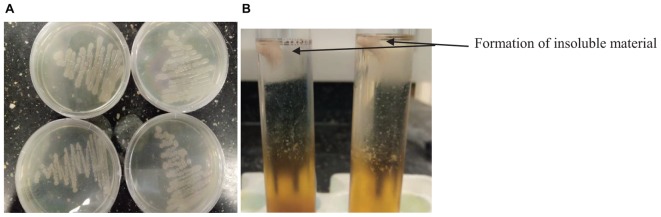
**(A)** Appearance of PGA-producing *Bacillus* spp. **(B)** and formation of insoluble material after addition of ethanol into PGA medium.

*Bacillus* species have been identified in various fermented food products. The growth of any microorganism in a fermenting medium may be influenced by initial inoculum, pH, type of fermentation, food substrate, temperature and nature fermenting microorganism (Karovičová and Kohajdova, 2007; Todorov and Holzapfel, 2015). In this study, the colony forming units of *Bacillus* spp. gradually decreased with an increase in fermentation time and varied with the type of cereal (Table 1). Although the number of *Bacillus* species was higher in OSL than LOSL, it was not significantly different (*p* > 0.05). After 18 h of fermentation, the growth of *Bacillus* spp. was not observed in LJOSL. However, *Bacillus* spp. was isolated in OSL (millet) at 48 h of fermentation. The varied colony forming units (CFU/ml) of *Bacillus* in fermented liquor suggest differences in the mechanisms of fermentation in each cereal. Findings are in agreement with the report of Okeke et al. (2015) that *Bacillus* species are dominant in all forms of *ogi* liquor at the early stage of fermenting but may gradually phase out as other competing microorganisms emerge. The gradual decrease of *Bacillus* spp. may be due to the progressive acidification of OSL and LOSL, growth of other LAB and yeast (Ijabadeniyi, 2007; Obi, 2014; Ajayeoba et al., 2016). Isolates were randomly selected from OSL and LJOSL (4 isolates from each category and at each fermenting intervals) and phenotypically characterized as *B. lichenformis, Bacillus subtilis, Bacillus cereus, Bacillus thuringienesis*, and *Bacillus amyloliquefaciens* respectively. A total of 40 isolates exhibited varying degrees of stickiness were observed at the early hours of fermentation (0–12 h). PGA-producing *Bacillus* spp. are potentially characterized with shiny, milk white and mucoid morphology (Li et al., 2008; Kolton et al., 2017) as shown in Figure 1A.

**Table 1:** Distribution of *Bacillus* species in *Ogi* steep liquor and lemon-*Ogi* steep liquor.

Fermentation time	WM-OSL	WS-OSL	Mi-OSL	CB-OSL	WM-LOSL	WS-LOSL	Mi-LOSL	CB-LOSL
0 h	5.50 ± 0.08^a^	5.23 ± 0.07^a^	5.40 ± 0.06^a^	5.32 ± 0.03^a^	5.50 ± 0.08^a^	5.23 ± 0.07^a^	5.40 ± 0.06^a^	5.32 ± 0.03^a^
6 h	5.24 ± 0.05^a^	4.96 ± 0.17^a^	5.22 ± 0.02^a^	5.00 ± 0.01^a^	5.19 ± 0.06^a^	4.87 ± 0.24^a^	5.18 ± 0.01^a^	4.93 ± 0.04^a^
12 h	4.15 ± 0.00^a^	3.94 ± 0.16^a^	5.01 ± 0.14^a^	3.74 ± 0.06^a^	3.78 ± 0.21^a^	3.39 ± 0.12^a^	4.03 ± 0.11^a^	3.65 ± 0.07^a^
18 h	3.74 ± 0.09^a^	2.95 ± 0.21^b^	4.04 ± 0.06^a^	3.20 ± 0.28^a^	3.39 ± 0.12^a^	3.00 ± 0.01^a^	3.97 ± 0.12^a^	3.15 ± 0.21^a^
24 h	3.09 ± 0.34^b^	2.34 ± 1.22^a^	3.25 ± 0.01^b^	3.04 ± 1.75^b^	0.00 ± 0.00	0.00 ± 0.00	0.00 ± 0.00	0.00 ± 0.00
36 h	2.17 ± 0.93^a^	0.00 ± 0.00	3.01 ± 0.41^b^	0.00 ± 0.00	0.00 ± 0.00	0.00 ± 0.00	0.00 ± 0.00	0.00 ± 0.00
48 h	0.00 ± 0.00	0.00 ± 0.00	2.10 ± 0.92	0.00 ± 0.00	0.00 ± 0.00	0.00 ± 0.00	0.00 ± 0.00	0.00 ± 0.00
56 h	0.00 ± 0.00	0.00 ± 0.00	0.00 ± 0.00	0.00 ± 0.00	0.00 ± 0.00	0.00 ± 0.00	0.00 ± 0.00	0.00 ± 0.00
72 h	0.00 ± 0.00	0.00 ± 0.00	0.00 ± 0.00	0.00 ± 0.00	0.00 ± 0.00	0.00 ± 0.00	0.00 ± 0.00	0.00 ± 0.00

*Values represented as mean ± SD (n = 3); for each column, different subscripts lowercase letters indicate significantly different at (P < 0.05). WM-OSL, White maize-ogi steep liquor; WS-OSL, White sorghum-ogi steep liquor; Mi-OSL, Millet-ogi steep liquor; CB-OSL, Combined -ogi steep liquor; WM-LJOSL, White maize lemon juice-ogi steep liquor; WS-LJOSL, White sorghum lemon juice-ogi steep liquor; Mi-LJOSL, Millet lemon juice-ogi steep liquor; CB-LJOSL, Combined lemon juice-ogi steep liquor.*

The PGA production of the isolates was tested at 25, 30, and 45°C (Table 2). The isolates formed an insoluble fibrous precipitate after addition of twice the volume of 95% ethanol into the PGA medium (Figure 1B). On the basis of high fibrous precipitate at 30°C and pH 7.5, and stickiness of >15 cm, 4 isolates of *Bacillus* spp. (TA004, TA006, TA011, TA012) were used for further analysis (Table 2).

**Table 2:** Screening of stickiness and PGA production of *Bacillus* species.

Microorganism	Strain code/source	Stickiness	PGA production (30°C)
*Bacillus subtilis* (*n* = 16)	TA001 (OSL)	9	+
	TA001(OSL)	11	++
	TA003 (LJOSL)	6	++
	TA004 (LJOSL)	23	+++
	TA005 (OSL)	3	+
	TA006 (OSL)	15	+++
	TA007 (OSL)	7	++
	TA008 (OSL)	13	++
	TA009 (OSL)	5	+
	TA010 (OSL)	21	++
	TA011 (OSL)	19	+++
	TA012 (OSL)	23	+++
	TA013 (OSL)	22	++
	TA014 (OSL)	21	+
	TA015 (OSL)	13	++
	TA016 (OSL)	13	+
*Bacillus licheniformis* (*n* = 5)	TA017 (OSL)	17	++
	TA018 (OSL)	22	+
	TA019 (OSL)	20	++
	TA020 (LJOSL)	6	++
	TA021 (OSL)	16	++
*Bacillus thuringienesis n* = 4	TA022 (LJOSL)	5	+
	TA023 (OSL)	6	++
	TA024 (OSL)	8	+
	TA025 (OSL)	5	++
*Bacillus amyloliquefaciens N* = 2	TA026 (LJOSL)	13	++
	TA027 (OSL)	12	++
*Bacillus cereus* (*n* = 13)	TA028 (OSL)	9	-
	TA029 (OSL)	6	-
	TA030 (LJOSL)	7	-
	TA031 (LJOSL)	6	-
	TA032 (LJOSL)	5	-
	TA033 (OSL)	4	-
	TA034 (LJOSL)	4	-
	TA035 (OSL)	8	-
	TA036 (LJOSL)	5	-
	TA037 (LJOSL)	4	-
	TA038 (LJOSL)	7	-
	TA039 (OSL)	3	-
	TA040 (OSL)	8	-

*N, number of isolates in parenthesis; +++, high clumping of insoluble precipitate; ++, more clumping of precipitate; +, moderate clumping of precipitate; -, no clumping of precipitate. No precipitate was observed at 25 and 45°C.*

### Functional Properties

#### ACE Inhibitory Assay

**FIGURE 2:**
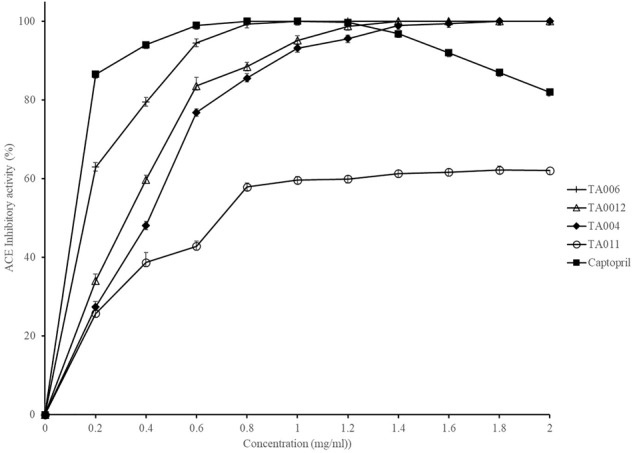
Angiotensin-converting enzyme (ACE) inhibition activity of γPGA and Captopril.

The ACE inhibitory activity of γPGA polymers and captopril (control) are shown in Figure 2. The activity was strain dependent but increased with increase in γPGA concentration. 100% inhibitory activity was observed at 1.00, 1.40, and 1.80 mg/ml for TA006, TA012, TA004 respectively, while TA011 did not attain 100% inhibition at the concentrations used in this study. The activity of Captopril was also concentration-dependent but reached 100% inhibition at 0.8 mg/ml and a gradual decline was observed afterward. The IC_50_ values (mg/ml) are TA004-0.35225; TA006-0.11512; TA012-0.28816; Captopril- 0.00534. ACE inhibition is a fundamental approach in the treatment of hypertension by regulating the renin-angiotensin system of blood pressure in humans. ACE inhibitory activities have been reported in a variety of fermented food, including cereal-based products (Hernández-Ledesma et al., 2004; Wang et al., 2016; Wu et al., 2018) and γPGA extracted from *Bacillus* spp. (Weng and Chen, 2012; Lee et al., 2014a,b). In addition to peptides that may be present in γPGA, ACE-inhibitory activity might be due to oligosaccharides and protease inhibitor that are associated with hydroxyl groups to establish hydrogen bonds with ACE (Pihlanto et al., 2008). The ACE activity of *B. amyloliquefaciens* (TA006) is comparable to earlier reported results (Weng and Chen, 2012; Lee et al. 2014a). Although the inhibitory activity and IC_50_ of Captopril were higher, we demonstrated that some *Bacillus* spp. in OSL and LOSL have ACE inhibitory potentials with possible application in reducing high blood pressure.

#### Antibacterial Activities

The antibacterial activities of the γPGA polymer are shown in Table 3. The paper disc diffusion assay showed varied zones of inhibition against each pathogen. Generally, the γPGA polymer of isolates TA004 and TA006 exhibited higher antimicrobial activity and the zones of inhibition (mm) were comparable to standard antibiotics. *S. aureus* and *L. monocytogenes* showed higher sensitivity than *E. coli, Salmonella enterica*, and *P. aeruginosa* while *Bacillus cereus* was completely resistant. Furthermore, the MIC varied with the type of γPGA (Table 3). TA004 showed the least MIC (16.125 mg/ml) against *S. aureus* while the highest MIC (>125 mg/ml) was observed in TA012 against *Salmonella enterica*. The resistance activity demonstrated by Gram-negative microorganisms (*Salmonella enterica* and *Pseudomonas aeruginosa*) is similar to previous reports (Lee et al., 2014a; Bajestani et al., 2018). The slight variation in MIC values, as compared to earlier reports, suggest differences in the isolate producing γPGA and test strains. Antimicrobial activity of γPGA is significantly influenced by the microbial source, charge, hydrophilic and anionic nature. Gram negative microorganisms are negatively charged (Bajestani et al., 2018) and environmental conditions can trigger rapid adaptive modification to enhance its survival. This is achieved by the release of outer membrane vesicles thus causing a significant increase in cell surface hydrophobicity and an enhanced tendency to form biofilms (Krasowska and Sigler, 2014), hence more concentration may be required to achieve a suitable reduction in bacterial population. The complete resistance of *Bacillus cereus* may be due to the biofilm matrix consists of structural proteins that form a hydrophobic envelope, assembling into an amyloid-like fibers network that strengthens the biofilm (Majed et al., 2016).

**Table 3:** Antibacterial and minimum inhibitory concentration (MIC) of γPGA polymers.

Source of γPGA	Concentration (mg/ml)/MIC value	*E. coli* ATCC 25922	*S. aureus* ATCC 29213	*P. aeruginosa* ATCC 27853	*L. monocytogenes* ATCC 7644	*B. cereus* ATCC 10876	*Salmonella enterica* ATTCC 13343
TA004	150	18.5 ± 0.71^∗^	22.5 ± 2.12	9.75 ± 0.35	21.65 ± 0.92	0.00 ± 0.00	14.15 ± 0.49
	MIC	≥32.25	≥16.125	≥125	≥32.25	ND	≥64.5
TA006	150	17.50 ± 0.71	18.95 ± 1.20	0.00 ± 0.00	20.00 ± 1.41	0.00 ± 0.00	15.60 ± 0.42
	MIC	≥32.25	≥32.25	ND	≥32.25	ND	≥64.5
TA011	150	12.40 ± 0.57	17.1 ± 0.57	0.00 ± 0.00	12.5 ± 0.71	0.00 ± 0.00	0.00 ± 0.00
	MIC	≥64.5	≥32.25	ND	≥64.5	ND	ND
TA012	150	12.8 ± 0.28	16.6 ± 1.56	0.00 ± 0.00	15.75 ± 1.06	0.00 ± 0.00	12.8 ± 0.42
	MIC	≥64.5	≥32.25	ND	≥64.5	ND	>125
Chloramphenicol		20.25 ± 0.35	19.45 ± 0.78	15.1 ± 0.14	19.9 ± 0.28	17.45 ± 0.35	16.95 ± 0.53
Ciprofloxacin		24.3 ± 0.14	26.75 ± 0.35	18 ± 0.14	15.5 ± 0.14	16.8 ± 0.42	18.05 ± 0.19

*Values are means ± standard deviation of duplicates in two trials. ND, Not determined. ^∗^Zone of inhibition.*

#### Kill-Time Kinetics

Table 4 shows the kill-time pattern of γPGA polymers against pathogenic microorganisms. Although there were significant reductions in the CFU/ml of each pathogen at 3 h, a slight increase in growth was observed at 6 h and thereafter. The initial decrease varied with the pathogen and type of γPGA used. Previous authors have similar reports on the increase in growth of pathogens at certain intervals after MIC exposure (Sim et al., 2014; Chen et al., 2016; Appiah et al., 2017). The log-reduction and subsequent regrowth suggest that the antimicrobial effect of γPGA is concentration and time-dependent. The regrowth may be attributed to distinct resistant sub-populations with varying susceptibilities, which take over the preferential killing of the susceptible sub-populations after a specified time of interaction (Sim et al., 2014). At 24 h, TA-004 achieved the highest log-reduction at 1xMIC (*S. aureus*- 2.35 log CFU/ml, *E. coli*- 1.97 log CFU/ml; *L. monocytogenes*- 2.24 log CFU/ml) while the lowest log- reduction was observed in TA011 (*S. aureus*- 1.42 log CFU/ml, *E. coli*- 1.60 log CFU/ml, *L. monocytogenes*- 0.87 log CFU/ml). At 2xMIC, a similar trend was observed in TA004 and TA011 (Table 4).

**Table 4:** Time-kill kinetics of γPGA polymers against pathogenic microorganisms.

Pathogenic microorganism	Control/Type of PGA	Time (h) and growth of pathogenic microorganism (log CFU/ml)									Log difference
		0	3	6	9	12	15	18	21	24	
*S. aureus*	Control	6.58 ± 0.91	7.25 ± 0.79	8.26 ± 0.31	8.32 ± 0.92	8.70 ± 1.02	8.70 ± 0.82	8.70 ± 0.45	8.70 ± 0.09	8.70 ± 0.34	
	TA004 (1xMIC)	6.59 ± 0.05	4.05 ± 0.24	4.06 ± 0.70	4.33 ± 0.78	4.69 ± 1.05	5.17 ± 0.58	5.79 ± 0.16	6.30 ± 0.87	6.35 ± 0.69	2.35
	TA004 (2xMIC)	6.59 ± 0.22	3.60 ± 0.88	3.62 ± 0.58	3.65 ± 0.97	3.80 ± 0.74	4.43 ± 0.92	4.96 ± 0.41	5.30 ± 0.17	5.77 ± 0.83	2.93
	TA006 (1xMIC)	6.59 ± 0.13	4.19 ± 0.45	4.22 ± 0.74	4.42 ± 0.81	5.59 ± 0.89	5.90 ± 0.66	6.02 ± 0.57	6.17 ± 0.53	6.57 ± 0.52	2.13
	TA006 (2xMIC)	6.59 ± 0.18	3.97 ± 0.21	3.90 ± 0.14	3.94 ± 0.49	4.67 ± 0.86	5.17 ± 0.54	5.83 ± 0.33	6.05 ± 0.64	6.12 ± 0.61	2.58
	TA011 (1xMIC)	6.63 ± 0.46	5.60 ± 0.84	5.81 ± 0.61	5.88 ± 1.03	6.00 ± 0.93	6.09 ± 1.08	6.15 ± 0.27	7.24 ± 0.75	7.28 ± 0.27	1.42
	TA011 (2xMIC)	6.63 ± 0.53	4.78 ± 1.20	4.62 ± 0.36	4.69 ± 0.73	5.02 ± 0.78	5.70 ± 1.12	6.20 ± 0.59	6.65 ± 0.44	6.65 ± 0.84	2.05
	TA012 (1xMIC)	6.66 ± 0.26	5.72 ± 0.37	5.72 ± 0.43	5.73 ± 0.22	5.81 ± 0.94	6.28 ± 0.92	6.57 ± 0.94	6.77 ± 0.42	6.98 ± 0.39	1.72
	TA012 (2xMIC)	6.66 ± 0.67	5.13 ± 0.41	4.99 ± 0.55	5.09 ± 0.71	5.44 ± 0.51	5.85 ± 0.98	6.16 ± 0.38	6.33 ± 0.75	6.33 ± 0.17	2.37
*E. coli*	Control	6.68 ± 0.45	7.01 ± 0.49	7.59 ± 0.91	8.27 ± 0.44	8.79 ± 1.41	8.79 ± 0.87	8.79 ± 0.84	8.79 ± 0.29	8.79 ± 0.13	
	TA004 (1xMIC)	6.57 ± 0.39	4.26 ± 0.51	4.42 ± 0.57	4.56 ± 0.21	5.68 ± 1.80	5.92 ± 0.91	6.32 ± 1.02	6.62 ± 0.16	6.82 ± 0.08	1.97
	TA004 (2xMIC)	6.57 ± 0.10	3.05 ± 0.08	3.01 ± 0.43	3.25 ± 0.37	4.67 ± 1.32	5.00 ± 1.00	5.64 ± 0.59	5.98 ± 0.33	6.30 ± 0.29	2.49
	TA006 (1xMIC)	6.66 ± 0.47	4.40 ± 0.13	4.78 ± 1.04	4.85 ± 0.19	5.86 ± 1.17	6.07 ± 0.57	6.66 ± 0.37	6.93 ± 0.23	7.02 ± 0.12	1.77
	TA006 (2xMIC)	6.66 ± 0.63	3.28 ± 0.42	3.36 ± 0.68	4.31 ± 0.28	4.91 ± 1.29	5.16 ± 0.49	5.85 ± 0.62	6.02 ± 0.41	6.58 ± 0.29	2.21
	TA011 (1xMIC)	6.64 ± 0.52	5.67 ± 0.37	6.09 ± 0.42	6.50 ± 0.16	6.74 ± 0.96	6.93 ± 0.66	7.00 ± 0.50	7.14 ± 0.36	7.19 ± 0.48	1.60
	TA011 (2xMIC)	6.64 ± 0.31	5.00 ± 0.31	5.19 ± 0.61	5.30 ± 0.88	5.87 ± 1.02	7.09 ± 0.89	7.09 ± 0.76	7.09 ± 0.81	7.09 ± 0.16	1.70
	TA012 (1xMIC)	6.70 ± 0.14	6.72 ± 0.18	6.73 ± 0.39	6.88 ± 0.41	7.11 ± 1.09	7.17 ± 0.95	7.26 ± 0.23	7.28 ± 0.27	7.29 ± 0.22	1.49
	TA012 (2xMIC)	6.70 ± 0.21	6.03 ± 0.29	6.04 ± 0.54	6.19 ± 0.32	6.42 ± 0.94	6.44 ± 0.76	6.57 ± 0.61	6.59 ± 0.34	6.60 ± 0.31	2.18
*L. monocytogenes*	Control	6.48 ± 0.32	7.39 ± 0.36	7.87 ± 0.86	8.65 ± 0.13	8.72 ± 1.03	8.72 ± 0.98	8.72 ± 0.46	8.72 ± 0.27	8.72 ± 0.39	
	TA004 (1xMIC)	6.67 ± 0.15	4.16 ± 0.46	4.18 ± 0.97	4.56 ± 0.89	4.78 ± 0.94	4.92 ± 0.76	6.44 ± 0.38	6.47 ± 0.82	6.48 ± 0.68	2.24
	TA004 (2xMIC)	6.67 ± 0.22	3.32 ± 0.28	3.13 ± 0.75	3.47 ± 0.57	3.91 ± 0.94	4.33 ± 1.24	4.64 ± 0.65	5.48 ± 0.46	5.93 ± 0.21	2.79
	TA006 (1xMIC)	6.66 ± 0.18	4.40 ± 0.16	4.78 ± 0.83	4.85 ± 0.72	5.86 ± 0.86	5.88 ± 0.81	5.94 ± 0.19	6.01 ± 0.21	6.57 ± 0.17	2.15
	TA006 (2xMIC)	6.66 ± 0.32	3.43 ± 0.25	3.31 ± 1.04	3.88 ± 0.94	3.99 ± 0.65	4.66 ± 0.55	5.30 ± 0.84	5.87 ± 0.30	6.01 ± 0.52	2.71
	TA011 (1xMIC)	6.64 ± 0.14	5.67 ± 0.36	6.89 ± 1.20	7.07 ± 0.82	7.18 ± 0.83	7.21 ± 0.79	7.71 ± 0.25	7.84 ± 0.18	7.85 ± 0.48	0.87
	TA011 (2xMIC)	6.64 ± 0.28	4.91 ± 0.47	4.92 ± 0.49	5.31 ± 0.67	5.42 ± 1.22	5.93 ± 0.96	6.47 ± 0.38	6.74 ± 0.28	7.06 ± 0.28	1.66
	TA012 (1xMIC)	6.70 ± 0.15	6.00 ± 0.52	6.23 ± 0.63	6.88 ± 0.51	7.11 ± 1.09	7.17 ± 0.49	7.55 ± 0.77	7.55 ± 0.35	7.55 ± 0.61	1.17
	TA012 (2xMIC)	6.70 ± 0.27	6.00 ± 0.22	6.01 ± 0.58	6.16 ± 0.47	6.62 ± 1.14	6.68 ± 0.80	6.88 ± 0.52	6.88 ± 0.79	6.88 ± 0.35	1.84
*S. aureus*	Control	6.37 ± 0.76	7.05 ± 0.18	7.75 ± 1.05	8.32 ± 0.87	8.98 ± 0.41	8.98 ± 0.34	8.98 ± 0.80	8.98 ± 0.26	8.98 ± 0.44	
	Combined (1xMIC)	6.85 ± 0.54	3.04 ± 0.35	3.19 ± 0.37	3.42 ± 0.52	4.94 ± 0.32	4.94 ± 0.21	4.98 ± 0.93	4.98 ± 0.50	5.00 ± 0.50	3.99
	Combined (2xMIC)	6.85 ± 0.44	2.89 ± 0.50	2.82 ± 0.49	2.97 ± 0.33	3.28 ± 0.29	3.88 ± 0.39	4.18 ± 0.54	4.31 ± 0.29	4.38 ± 0.27	4.60
*L. monocytogenes*	Control	6.67 ± 0.51	7.14 ± 0.86	7.82 ± 0.91	8.20 ± -.12	8.88 ± 0.53	8.88 ± 0.27	8.88 ± 0.39	8.88 ± 0.44	8.88 ± 0.95	
	Combined (1xMIC)	6.79 ± 0.18	3.99 ± 0.67	3.92 ± 0.84	4.02 ± 0.70	4.21 ± 0.45	4.31 ± 0.27	4.40 ± 0.44	4.93 ± 0.71	4.93 ± 0.89	3.95
	Combined (2xMIC)	6.79 ± 0.31	3.65 ± 0.25	3.78 ± 0.43	3.79 ± 0.46	3.81 ± 0.36	3.93 ± 0.41	4.20 ± 0.70	4.32 ± 0.32	4.36 ± 0.70	4.52
*E. coli*	Control	6.47 ± 0.46	7.24 ± 0.41	7.92 ± 0.70	8.56 ± 0.27	9.11 ± 0.24	9.11 ± 0.85	9.11 ± 0.71	9.11 ± 0.45	9.11 ± 0.19	
	Combined (1xMIC)	6.69 ± 0.70	4.09 ± 0.28	4.09 ± 0.56	4.09 ± 1.01	4.32 ± 0.69	4.53 ± 0.17	4.66 ± 0.25	4.89 ± 0.18	5.43 ± 0.34	3.68
	Combined (2xMIC)	6.69 ± 0.69	4.01 ± 0.13	4.01 ± 0.68	4.01 ± 0.88	4.01 ± 1.34	4.19 ± 0.24	4.36 ± 0.38	4.78 ± 0.69	4.98 ± 0.23	4.13

The combined γPGA demonstrated a higher log reduction pattern at 1xMIC (*S. aureus*- 3.99 log CFU/ml, *E. coli*- 3.68 log CFU/ml; *L. monocytogenes*- 3.95 log CFU/ml) and 2xMIC (*S. aureus*- 4.60 log CFU/ml, *E. coli*- 4.13 log CFU/ml; *L. monocytogenes*- 4.52 log CFU/ml) than single use. Time-kill curves have been used to determine the pharmacodynamics of substance in-vitro (Ferro et al., 2015). Therefore, the individual log-reduction pattern obtained in this study are bacteriostatic (<3.0 log), while the combined forms exhibited bactericidal effects (>3log).

### Characteristics of γPGA Polymer

The yield, protein and polysaccharide contents of the γPGA varied with the type of isolate. However, the molecular weights of γPGA polymers (Table 5) showed that TA004 had the highest (261.44 kDa) while TA011 had the least (194.97 kDa). The addition of ethanol to the broth supernatant used in growing γPGA-producing *Bacillus* is known to precipitate polysaccharides and proteins (Buescher and Margaritis, 2007). The polysaccharide and protein components are the major constituent of γPGA matrix that influences its functionality. The protein nature of γPGA determines the economic and environmental application of the polymer (Ho et al., 2006). The molecular weight of γPGA was determined by diffusion distances (TA 004–10.43 mm; TA006-10.47 mm; TA011-10.48 mm; TA012-10.44 mm) in agar plate as shown in Table 5.

**Table 5:** Analysis and quantification of γPGA.

Type	Analysis of PGA		Quantification of PGA	
	Protein content (%)	Polysaccharide content (%)	Yield (%)	Molecular weight (kDa)
TA 004	14.31 ± 0.10	1.04 ± 0.16	9.09 ± 0.78	261.44
TA006	10.97 ± 0.12	1.34 ± 0.30	7.72 ± 1.16	202.36
TA011	6.74 ± 0.13	1.54 ± 0.24	29.11 ± 0.91	194.97
TA012	9.31 ± 0.03	1.32 ± 0.39	16.86 ± 1.42	254.06

Microbial production of γPGA can have molecular weights ranging from 100 to over 2,000 kDa (Ho et al., 2006; Ogunleye et al., 2015; Luo et al., 2016). The yield and molecular weight of PGA are dependent on nature and pH the substrate, microbial species involved, composition, cultivation conditions, and time of fermentation (Markovsky et al., 2012; Lee et al., 2018). These influences the specificity (Ogunleye et al., 2015) and explains the importance of the substrate to isolate PGA-producing *Bacillus* spp. Although potent ACE-inhibitory compounds reported were released from fermented protein-based products (Chettri et al., 2016), this research shows that other fermented products may have ACE inhibitory compounds in some *Bacillus* spp., depending on the fermenting conditions. Furthermore, the physicochemical characteristics of the polymers, such as molecular weight, charge, chemical composition and polydispersity influences the properties and applications of a polymer (Markovsky et al., 2012). Most of the ACE inhibitors known for drug delivery have low molecular-weight-compounds, usually between 30 and 50 kDa (Lee et al., 2018) because it does not readily diffuse through normal capillaries and glomerular endothelium, therefore additional downstream purification steps will be necessary to isolate specific molecular weight fractions from γPGA used in this study (Richard and Margaritis, 2001). Antimicrobial properties are desired attribute of a substance intended for food and biomedical applications. There are few reports on the antimicrobial functions of pure γPGA. Although it is reported that higher molecular weight of polymer may diffuse slowly, the factors that are considered for antimicrobial activity are surface charge, hydrophobicity, hydrophilicity, and intramolecular hydrogen bonding (Khalil et al., 2017; Wang et al., 2017), and these differ between producing strains.

#### Fourier Transform Infra-Red Analysis of γPGA

**FIGURE 3:**
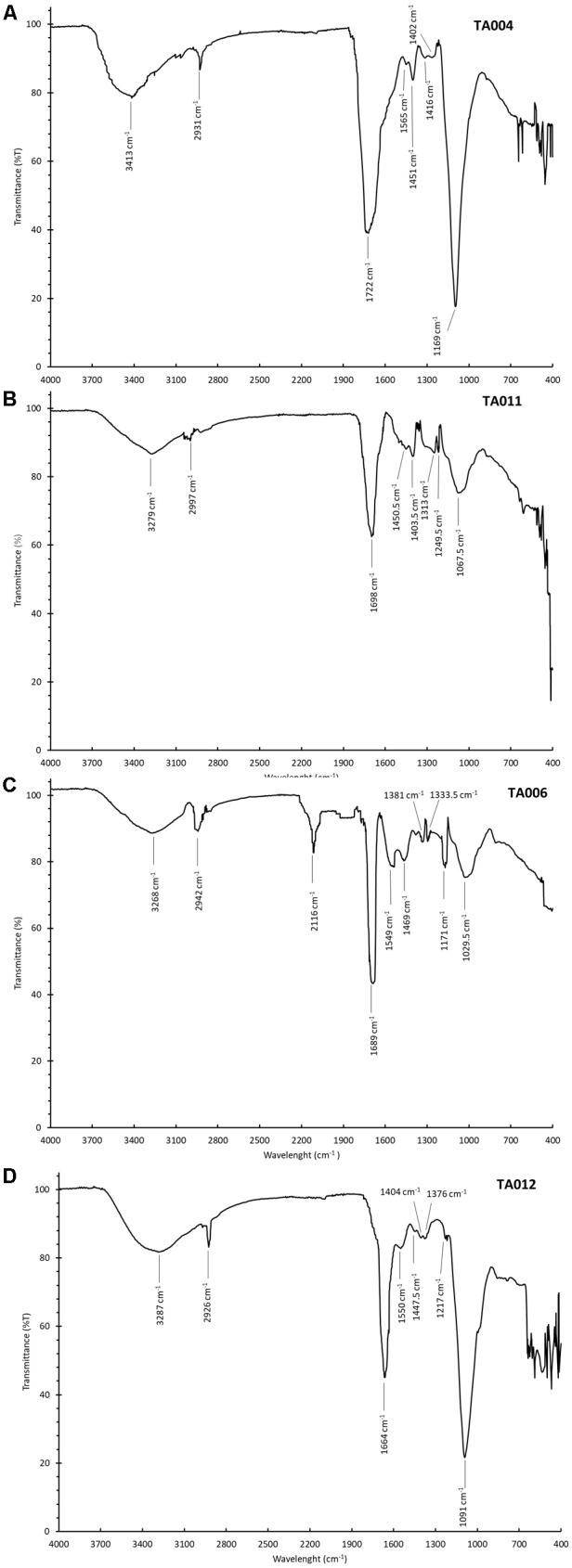
Fourier Transform Infra-Red Spectroscopy (FTIR) Spectra of γPGA polymers. **(A)** TA004, **(B)** TA011, **(C)** TA006, and **(D)** TA012.

Figure 3A–D shows the FTIR spectra of the γPGA. The spectra had peaks around the same range. The broad peak at 3413–3268 cm^-1^ corresponded to amine N–H stretching intensities while the peak observed within the range of 2997–2926 cm^-1^ is attributed to hydrogen bonding. The sharp peaks around 1722–1664 cm^-1^ depicts the C = O stretching in COOH. The other peaks thereafter represent C = O and C-N groups respectively. The peaks of FTIR spectra observed in this study correspond to similar peaks reported by Ogunleye et al. (2015).

### *16s rRNA* Sequencing of γPGA-Producing Isolates

**FIGURE 4:**
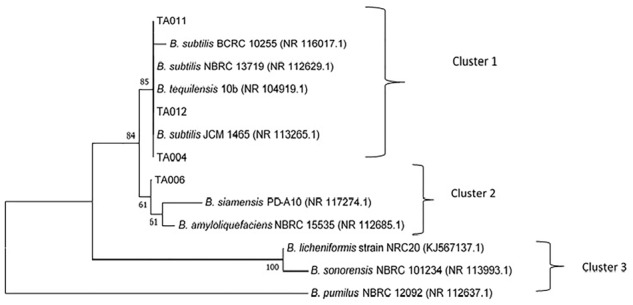
Evolutionary relationships of the analyzed isolates with their closest known taxa.

The accession numbers of *Bacillus* spp. identified in this study are TA004 (*B. subtilis*-GenBank:MH782061), TA006 (*B. amyloliquefaciens*- GenBank:MH782075), TA011 (*B. subtilis*- GenBank:MH782088), TA012 (*B. subtilis*- GenBank:MH782083). The confidence degree, *E* = 0.0 and homology similarity are between 99 and 100% for all the isolates. The phylogenetic tree constructed with neighbor-joining method was based on the evolutionary relationships and three distinct clusters were formed (Figure 4) *Bacillus subtilis* (TA004, TA011, TA012) showed 85% similiarity with *B. subtilis* JCM 1465, *B. tequilensis* 10b, *B. subtilis* NBRC 13719 and *B. subtilis* BCRC 10255 as shown in cluster 1. TA006 (*Bacillus amyloliqueficiens*) was observed in same clade showing 61% similarities with *B. siamensis* PD-A10 and *B. amyloliquefaciens* NBRC 15535 among others. *B. subtilis* and *B. amyloliquefaciens* are known γPGA producers (Chettri et al., 2016; Luo et al., 2016). These relationships suggest that some *Bacillus* spp. identified in *Ogi* steep liquor could have possible application in industrial settings.

## Conclusion

γPGA-producing *Bacillus* spp. were isolated during fermentation of OSL and LOSL at 0–12 h. These isolates exhibited antibacterial and ACE inhibitory activities at different concentrations. The individual antibacterial activities of γPGA were bacteriostatic while the combined use exhibited bactericidal characteristics. Although the ACE inhibitory activities were low, OSL and LOSL are promising sources for developing functional microorganisms with a beneficial impact on health. The importance lies in the benefits derived from the use of local resources.

## Author Contributions

TA: contributed to the conceptualization of the study, the screening of PGA-producing Bacillus from different OSL and LJOSL, the phenotypic identification, the stickiness, and performed the screening of PGA. TA contributed to the antibacterial and ACE inhibitory activities, time-kill kinetics of PGA, prepared the draft of the manuscript and performed the analysis of the data. SD: performed the FTIR analysis of PGA, and the antibacterial activities of PGA. OAI: contributed to the conceptualization of the study and the compilation and the finalization of the manuscript.

## Conflict of Interest Statement

The authors declare that the research was conducted in the absence of any commercial or financial relationships that could be construed as a potential conflict of interest.

## Footnotes

1 http://blast.ncbi.nlm.nih.gov/Blast.cgi
